# Measuring psychopathology as it unfolds in daily life: addressing key assumptions of intensive longitudinal methods in the TRAILS TRANS-ID study

**DOI:** 10.1186/s12888-020-02674-1

**Published:** 2020-07-06

**Authors:** Marieke J. Schreuder, Robin N. Groen, Johanna T. W. Wigman, Catharina A. Hartman, Marieke Wichers

**Affiliations:** University of Groningen, University Medical Center Groningen, Interdisciplinary Center Psychopathology and Emotion regulation, Groningen, The Netherlands

**Keywords:** Daily diary studies, Transdiagnostic psychopathology, At risk mental state, Intensive longitudinal methods, Personalized designs, Young adulthood, Feasibility

## Abstract

**Background:**

Intensive longitudinal (IL) designs provide the potential to study symptoms as they evolve in real-time within individuals. This has promising clinical implications, potentially allowing conclusions at the level of specific individuals. The current study aimed to establish the feasibility of IL designs, as indicated by self-rated burden and attrition, in the context of psychiatry. Additionally, we evaluated three core assumptions about the instruments (diary items) used in IL designs. These assumptions are: diary items (1) reflect experiences that change over time within individuals (indicated by item variability), (2) are interpreted consistently over time, and (3) correspond to retrospective assessments of psychopathology.

**Methods:**

TRAILS TRANS-ID is an add-on IL study in the clinical cohort of the TRAILS study. Daily diaries on psychopathological symptoms for six consecutive months were completed by 134 at risk young adults (age 22.6 ± 0.6 years). At baseline, immediately after the diary period, and one year after the diary period, participants completed a diagnostic interview.

**Results:**

Excellent compliance (88.5% of the diaries completed), low participant burden (M = 3.21; SD = 1.42; range 1–10), and low attrition (8.2%) supported the feasibility of six-month IL designs. Diary items differed in their variability over time. Evaluation of the consistency of diary item interpretations showed that within-individual variability in scores could not be attributed to changing interpretations over time. Further, daily symptom reports reasonably correlated with retrospective assessments (over a six month period) of psychopathology obtained with the diagnostic interview, suggesting that both measures might complement each other.

**Conclusion:**

The current study is the first to show that IL designs over extensive periods (i.e., multiple months) in psychiatry are feasible, and meet three core assumptions to study change in psychopathology. This might allow for addressing novel and promising hypotheses in our field, and might substantially alter how we treat and study mental ill-health.

## Background

Symptoms of mental ill-health are inherently time-varying: they may progress from mild complaints to severe disorders and vice versa [1]. This may play out over longer periods, such as months or even years, but also can occur much more rapidly over the span of several days [2] (Wichers M, Smit AC, and Snippe E: Early warning signals based on momentary affect dynamics can expose nearby transitions in depression: a confirmatory single-subject time-series study, submitted). Traditional longitudinal designs, which generally assess large groups of individuals a few times over long time-intervals, are capable of investigating slower changes at the group level such as developmental changes (e.g. developmental differences between typically and non-typically developing children). However, these designs cannot provide insight at the individual level, e.g. whether a particular individual is likely to develop anxiety symptoms in the near future, or conversely, is likely to remit. In order to address questions relevant to the individual patient, we need to examine how daily experiences of a specific individual change over the course of several months. Such designs – hereafter referred to as intensive longitudinal (IL) designs – are rigorous but necessary, as they will enable us to connect macro-level changes in individuals (exposed by traditional longitudinal designs) with micro-level processes, such as their vulnerability to mental ill-health at a particular moment in time.

IL designs accommodate several complex features of psychopathology that cannot be accounted for otherwise [1, 3, 4]. A first feature is that symptom manifestation and illness progression seem highly person-specific. Individuals differ from other individuals: even when diagnosed with the same disorder (e.g. major depression), individuals typically show marked differences in terms of the specific symptoms they experience and on what timescale they come and go [5]. Further, individuals differ from themselves over time: symptoms fluctuate within individuals both in severity and type (e.g. anxiety symptoms may substitute depressive symptoms, or vice versa) [6–8]. Fluctuations in severity may entail disorder onset, remission or relapse, and may also occur on a smaller scale, for instance between ‘mild’ and ‘moderate’ levels of severity [9, 10]. These person-specific features cannot be studied through traditional longitudinal designs, which is in part due to the relatively small number of repeated measurements (i.e. usually less than ten) in such studies. In contrast, IL designs such as diary or ecological momentary assessment studies easily yield over 50 time points per individual. This high number and granularity of measurements allows for characterizing the within-individual process, and thus provides the possibility to gain insight in core questions of psychiatry, for instance why some individuals develop symptoms, while others do not, and why some individuals experience a mild symptom course whereas others deteriorate over time.

When monitoring the development of psychopathology, a second feature is that assessments should be acquired during the period in which changes in symptomatology occur. For instance, in order to develop measures that enable detection of upcoming changes in symptoms, or gain insight in how switches in type of symptoms occur, the process of change needs to be observed while it unfolds. Instead of static snapshots with relative long time intervals in between as in traditional longitudinal designs (e.g. every 12 months), IL designs yield detailed insight into how symptoms change from day to day or moment to moment [4]. As symptom changes may involve both the type of symptoms that manifest and their severity, it is important to assess symptoms of various diagnostic categories simultaneously on a continuous scale. By allowing for variability between and within individuals in symptom profiles, IL designs may enable us to capture individual trajectories from subthreshold symptoms to full-blown disorders as well as transitions that occur between disorders.

As technology advances, the implementation of personalized designs has become increasingly feasible. For instance, IL data can be collected through electronic questionnaires which can be delivered via smartphones, making it possible to lengthen the measurement period. The latter is particularly important when studying how symptoms develop over time, as one needs to maximize the possibility of observing changes in symptoms. Despite increasing availability of methods to collect IL data, prospectively monitoring psychopathological symptom development through intensive designs with extended duration (>three months) has rarely been pursued before [11]. It is therefore important to investigate the feasibility of these designs. Thus, the first aim of this paper is to establish whether prospectively assessing symptoms through a six-month diary study is feasible. Specifically, participant burden – indicated by drop-out and non-compliance – will be evaluated. Second, because IL data show great promise in being able to capture symptom development, it is important to evaluate some of the core assumptions underlying the instruments used in these designs. In the current study we evaluate three such assumptions about daily diary items: 1) diary items are able to detect fluctuations in symptoms, 2) the interpretation of diary items remains consistent over time and 3) diary items reflect the same constructs that are measured with traditional instruments (e.g. retrospective diagnostic interviews).

These assumptions may call for some elaboration. The first assumption states that diary items are suitable for detecting fluctuations in respective symptoms. However, it is important to evaluate which items may be particularly well-suited for monitoring changes in specific symptom domains or populations. For instance, diary items that are appropriate for specific clinical samples (e.g., “To what extent did you hear voices today?” In those at high risk for psychosis) may not be suitable for samples from the general population or transdiagnostic samples with high risk for more common types of psychopathology, and vice versa. The second assumption involves the consistency of items over time. Because IL designs have long durations and require frequent introspection, it is possible that participants change their interpretation of diary items over the course of the study. For instance, feeling down might initially be perceived as intense sadness, and later be regarded as a mild sense of stress. Such a change makes interpreting the variability in feeling down over time difficult. While varying item interpretations can also occur in short-lasting designs, e.g. where symptoms are sampled for one or two weeks, the probability of changing interpretations increases with study duration. Hence, in the interest of the current as well as future IL designs, the time-invariance of diary item interpretations should be verified. Finally, given that both methods tap into psychopathology, a third assumption concerns the correspondence of symptom reports in the diary study to symptom reports in conventional assessments of psychopathology such as diagnostic interviews. In other words, we assume that the results of a diagnostic interview – which concern macro-level experiences based on retrospective assessments of psychopathological symptoms during several months – map to some extent onto micro-level experiences based on self-reports of symptoms in daily life. Analyzing this correspondence could potentially reveal which diary items are most suitable for detecting individual differences in specific diagnostic domains. In sum, the present paper will investigate whether intensive longitudinal assessments of psychopathology are feasible and whether the instruments in those designs (diary items) 1) pick up variability, 2) are consistently interpreted over time, and 3) correlate with macro-scale assessments (retrospective diagnostic interview). This may pave the way for the use of IL designs to address important questions in the field of psychopathology.

The current paper will address the above aims using data from a six-month daily diary study in young adults at risk for psychopathology. The study, TRAILS TRANS-ID, was designed to monitor the day to day fluctuations in psychopathological symptoms of participants from a clinical cohort as they entered young adulthood, which is considered a particularly vulnerable period in terms of mental ill-health (see TRAILS TRANS-ID infrastructure) [12, 13]. For six consecutive months, participants completed daily assessments concerning 58 symptoms of psychopathology. The TRAILS TRANS-ID study has not been described before, and we will therefore provide a detailed description of its methods. Ultimately, the resulting data might allow for conclusions that could considerably advance our understanding of how, why and when symptoms evolve.

## Methods

### TRAILS TRANS-ID infrastructure

TRAILS TRANS-ID connects two large, ongoing projects, namely TRAILS (TRacking Adolescents’ Individual Lives Survey) and TRANS-ID (Transitions In Depression^1^). TRANS-ID was designed to prospectively monitor sudden transitions in psychopathology. TRAILS TRANS-ID is one of the studies within the TRANS-ID project, and includes participants from TRAILS. TRAILS is an ongoing, prospective cohort study investigating mental health across pre-adolescence and young adulthood. TRAILS consists of a general population cohort (TRAILS-PC, which started in 2000) and a transdiagnostic clinical cohort (TRAILS-CC, which started in 2004). Participants of TRAILS TRANS-ID were recruited in TRAILS-CC. TRAILS-CC was designed to enrich its general population counterpart by selectively sampling individuals who were at heightened risk for mental ill-health. Inclusion in TRAILS-CC was based on at least one referral before the age of 11 years to the child psychiatric outpatient clinic of the University Medical Center Groningen. Because of this history, TRAILS-CC participants are considered vulnerable for developing (additional) psychopathological complaints later in life (e.g. in young adulthood). This is reflected in heightened mental ill-health in TRAILS-CC compared to the general population cohort of TRAILS [14]. An extensive description of the sampling procedures for TRAILS-CC has been published elsewhere [15].

### Study design

TRAILS TRANS-ID consisted of a six-month daily diary study that was designed to capture the ebb and flow of (subthreshold) symptoms of psychopathology in young adults at heightened risk for mental ill-health (see Study population). Immediately before (baseline), directly after (post assessment), and one year after the diary period (follow-up), a clinical interview took place. This interview included a diagnostic interview and two questionnaires on social functioning and life events, respectively. All measures were administered orally and are outlined in detailed below. All assessments and other contact moments with participants (e.g. phone calls) were performed by trained researchers (first two authors and research assistant) in a standardized way. Participants remuneration included up to a maximum of €200,- (for details, see Additional file 1) and a report of their own diary data.

### Study population

All participants enrolled in TRAILS-CC who participated in at least one of the previous measurement waves of TRAILS-CC and had given consent to be approached for future assessments were eligible to participate in the TRAILS TRANS-ID study (see also TRAILS TRANS-ID infrastructure). At its first measurement wave, TRAILS-CC included 543 children aged between 10 and 12 years old (mean age = 11.1, std. = 0.50, range 10.1–12.4, 34.1% girls). TRAILS TRANS-ID took place when the TRAILS-CC participants were between 21 and 24 years old. Of the 443 TRAILS-CC participants eligible for participation in TRAILS TRAILS-ID, 134 (30.2%) enrolled in the study (see details on recruitment below). The TRAILS-CC cohort study was approved by the Dutch Central Committee on Research Involving Human Subjects (CCMO), in accordance with the ethical standards laid down in the 1964 Declaration of Helsinki. Prior to each measurement wave of TRAILS, informed consent was obtained from the adolescents (at earlier waves also from the parents). The TRAILS TRANS-ID study was approved by the standing Ethics Committee, in accordance with the 1964 Declaration of Helsinki. All participants gave written informed consent before enrolment in TRAILS TRANS-ID.

### Procedure

#### Recruitment

From 18 October 2017 until 12 February 2018, individuals of the TRAILS-CC cohort were invited to participate in TRAILS TRANS-ID. Individuals received an invitation letter by mail, which contained information about the aims and design of TRAILS TRANS-ID. Individuals could indicate whether they were interested in participation through a website, by phone, or by returning the reply card that was enclosed to invitation letter. Participants who did not respond received a reminder by mail (approximately one month after the first invitation).

Upon registering, participants were contacted by phone by a researcher who provided further information about the study. This contact moment was also used to verify whether participants owned a smartphone and whether they anticipated any circumstances that might interfere with the diary period (e.g. long-term stay in remote areas). Participants who did not own a smartphone were provided one for the duration of the diary period. Of the 142 participants who were interested in participating in TRAILS TRANS-ID, 8 participants (5.6%) were not included for various reasons (e.g. because they could not be reached or had changed their minds).

#### Baseline assessment

Prior to the clinical interview, participants were once more informed about the study procedures and asked for written informed consent. Thereafter, the clinical interview (see Instruments) was conducted and the diary procedure was explained. Participants completed a practice session in presence of a researcher in order to familiarize themselves with the questions. Participants were instructed to carefully consider their mental state and use the extreme ends only if they could not imagine experiencing a certain symptom more (corresponding to a score of 100) or less (corresponding to a score of 0). To verify whether participants’ interpretation of diary items remained consistent during the diary period – as outlined in our second aim – half of the participants were interviewed about their interpretation of a randomly selected set of ten items after the clinical interview (see Qualitative assessment).

#### Diary period

Participants started the diary period immediately after the baseline assessment (Fig. S1, Additional file 1). This period comprised filling in an electronic diary every evening for six consecutive months (i.e. 183 days). Participants could access the diary via a link sent in a text message to their mobile phone. The diary consisted of questions assessing experiences during the past day (for an overview of items, see Table S3, Additional file 1). Participants therefore always received text messages in the evening, at a fixed time according to the participant’s wishes. Although timing of the text messages differed between participants, all participants had 24 h between each measurement point. Participants were asked to complete the questionnaire as soon as possible after receiving the text message. If the questionnaire was not completed within 30 mins, participants received a reminder text message. After this reminder, participants had 2.5 h to fill in the diary. The questionnaires were administered and stored via Roqua (www.roqua.nl).

During the diary period, the researchers contacted the participants at least seven times. The first two phone contacts took place in the first and second week of the study, respectively, to resolve any issues regarding, for instance, the timing of the text message or answer questions about the study procedures. Thereafter, participants were contacted monthly or when they skipped diaries for several consecutive days. These phone contacts served to support participants, to solve any issues that occurred (e.g. technical problems), and to monitor life events that might affect symptom dynamics. The latter were assessed by asking participants whether they had experienced important (positively or negatively appraised) events regarding their health, work or education, social relations, and financial situation. These events were noted and, together with the participant, retrospectively dated. Researchers were also available by telephone and email if participants needed assistance at other times than the pre-specified contact moments.

#### Post and follow-up assessment

After the diary period, participants were invited for a post assessment, which involved a clinical interview spanning the diary period (i.e. the past six months) and an evaluation of the diary. The latter included rating the burden of completing the diary period on a scale from one (not at all burdensome) to ten (extremely burdensome). Participants were offered a report on their personal data including descriptive information which was explained orally and provided on paper. Those who were included in the qualitative assessment of diary items at baseline, were asked to take part in this assessment again at post (see Qualitative assessment). Finally, one year after finishing the diary period, participants were invited for the follow-up assessment. This assessment involved a clinical interview (mini-SCAN, GVSG, life events questionnaire) that spanned the past year.

#### Diary measures

The diary questions, listed in Table S3 (Additional file 1), were selected to cover symptoms of all common psychiatric disorders. Items were derived from earlier studies that adopted ambulatory assessments or diary protocols [16–19] as well as from existing questionnaires such as the Positive and Negative Affect Scale (PANAS) [20], the Adult Behavior Checklist (ABCL) [21], and the Symptom Checklist (SCL-90) [22]. Items were considered for inclusion in the diary if they (1) covered multiple diagnostic categories; (2) were sufficiently distinct from other items; (3) were expected to vary from day to day; and (4) were easy to understand. To accomplish the latter, we conducted a pilot study among 12 adolescents with a similar educational attainment level to that of the TRAILS-CC cohort (see Additional file 1 for more information). The final selection consisted of 58 items, which were all rated on a visual analogue scale (VAS) that ranged from 0 to 100. Participants were given the opportunity to add an item to the diary that they personally wished to monitor (e.g. ‘To what extent was I productive at work today?’). At the end of the diary, an optional textbox allowed participants to make notes about their day. Participants were encouraged to report unusual or important events, such as starting a new job or being ill, which might have influenced their responses to the diary that day. Participants’ notes remained confidential and were solely examined during the post assessment for the descriptive report on their personal data.

#### Qualitative assessment: validity of diary items

To evaluate the validity of diary items – as indicated by the consistency of participants’ interpretation – the first 60 participants who were included completed an interview about their interpretation of a randomly selected set of ten items both at the baseline and post assessment. We interviewed 60 individuals rather than the whole sample because this was considered to yield a reliable indication of interpretation consistency, without needlessly burdening participants. During the qualitative assessment, participants were asked what a certain item, such as feeling stressed, meant to them. Participants were asked about the same set at baseline – when participants practiced with the diary – and during the post assessment – when participants discussed their personal data with the interviewer. At both assessments, participants’ interpretations were recorded and later transcribed. Participants were encouraged to elaborate and to provide examples. In case participants did not understand an item during the first session, the item’s meaning was explained by the researcher. In this case, consistency in interpretation was not evaluated for this item.

### Instruments

#### Demographic characteristics

Demographic characteristics of the sample, including socio-economic status and current occupation (education, employment), were retrieved from earlier measurement waves of the clinical cohort of TRAILS. Socio-economic status consisted of a standardized score that combined information about household income and the education and occupation of participants’ parents, and was assessed during the first measurement wave when participants were approximately 11 years old.

#### Diagnostic interview

The clinical interview that was administered at baseline, post assessment, and follow-up consisted of a diagnostic interview complemented by orally administered questionnaires on social functioning and life events (see below). The diagnostic interview consisted of the computer-assisted mini-SCAN which was administered by trained interviewers and assessed the presence of psychiatric disorders [23]. The mini-SCAN is a validated semi-structured interview, which by means of screening questions and skips efficiently assesses whether individuals meet the diagnostic criteria of a broad range of psychiatric disorders. Since current purposes were primarily of scientific, as opposed to traditional diagnostic, nature, we slightly adapted the assessment procedure (see also Table S4, Aditional file 1). First, similar to an earlier study [24], we rated items on a three-point scale (absent, subthreshold, clinical) rather than a dichotomous scale (absent, present). This allowed for recognizing symptoms that were endorsed by participants, but did not meet the criteria of clinical significance (e.g. with respect to severity, duration, or frequency; see Additional file 1 for details on scoring). For instance, feeling down for multiple consecutive days, but less than two weeks, could classify as a subthreshold symptom of depression. The distinction between absent, subthreshold and clinical symptoms was considered particularly relevant since our participants were at risk for psychopathological symptoms. This at risk status may frequently coincide with mild signs of psychopathology that occupy the grey area between mental health and psychopathology. Adapting the conventional rating procedure of the mini-SCAN allowed for capturing this grey area. A second adaptation concerned the removal of skips that are traditionally imposed by the mini-SCAN. To be able to compare ratings across but also within individuals, we assessed the following sections in each participant: stress, anxiety (including post-traumatic stress disorder (PTSD), generalized anxiety disorder (GAD), panic disorder, social anxiety disorder, specific phobia), obsessive-compulsive disorder (OCD), depression, mania, hallucinations, delusions, substance use, attention deficit hyperactivity disorder (ADHD), autism spectrum disorder (ASD). For the majority of sections, all symptoms were rated regardless of the presence of hallmark symptoms (e.g. for depression these are depressed mood and/or loss of interest). Sections for which skips were preserved were anxiety, hallucinations, and delusions, which all contained symptoms that were conditional upon the hallmark symptom(s). For instance, symptoms of PTSD are conditional upon the experience of a traumatic event and were hence disregarded in the absence of such an event.

The mini-SCAN does not cover oppositional or antisocial behavior, yet such behavior may represent an important precursor or manifestation of psychopathology [25]. Therefore, we complemented the mini-SCAN with the aggressive behavior subscale of the Adult Self Report (ASR, also assessed at earlier measurement waves of TRAILS; 17)), which was orally administered. This subscale consists of 15 items that are rated on a three-point scale (never or seldom, sometimes, frequently or always). Scores on the aggressive behavior subscale were considered indicative of symptoms of oppositional defiant disorder (ODD) and will henceforward be referred to as such.

#### Social functioning

Social functioning was assessed with the Groningen Social Behaviour Questionnaire (in Dutch: Groningse Vragenlijst Sociaal Gedrag, GVSG), which has good psychometric properties [26]. The GVSG consists of 45 items that assess functioning in several domains, including social relations (i.e. with parents, children, friends, and romantic partners), work, school, household, and leisure-time. Items were rated on a 4-point Likert scale which ranged from ‘never’ to ‘always’. Domain scores that exceeded the upper limit cut-off provided by De Jong and Van der Lubbe (2001, [26]) were categorized as ‘severe impairments in functioning’ and domain scores that exceeded the lower limit cut-off were categorized as ‘mild impairments in functioning’.

#### Life events

The life events questionnaire comprises an adapted version of the List of Threatening Experiences, which has good psychometric properties [27, 28]. The List of Threatening Experiences consists of 12 items describing major stressful life events, such as experiencing serious illness, losing a job, or ending a steady relationship. Eight items describing pleasant events, such as going on a holiday or graduating, were added to this list, resulting in a total of 20 items. The occurrence of these events (yes/no) was rated.

### Statistical analyses

The data presented in the current paper pertain to the baseline assessment, diary period, and the post assessment. Follow-up interviews were still being conducted. Analyses were conducted in R (version 1.463).

#### Aim 1: feasibility

Our first aim was to explore whether conducting a six-month daily diary study in at risk young adults is feasible. Feasibility was evaluated by examining descriptives (mean, standard deviation) concerning participants’ drop-out and compliance, and their responses to the diary-evaluation questions in the post assessment interview, which inquired about the burden and reasons for missing diary responses.

#### Aim 2: three core assumptions

Our second aim was to evaluate three common assumptions of IL assessments of psychopathology. The first assumption is that diary item capture variability in symptom expression. This assumption was evaluated by computing the between- and within-individual variability in symptom reports. Between-individual variability was operationalized as the deviance in mean item scores across individuals. Large values indicate that individuals differ considerably in their scores on the respective item, while small values suggest that individuals score relatively similar. Within-individual variability was examined through calculating the mean deviation in item scores within individuals. Large values indicate that the respective item varies considerably over time within individuals, while small values suggest that scores are relatively stable. As there are no criteria yet for the minimum amount of variability that can be considered informative, we compared different items descriptively.

The second assumption to evaluate was the consistency of participants’ interpretations of diary items over time. This was addressed by comparing, per participant and per item, interpretations at baseline and at post assessment. Item interpretations were coded as consistent when participants used identical or similar words or examples to describe an item at baseline and post assessment, respectively. Inconsistent interpretations, in contrast, were scored in case the interpretations at baseline and post assessment showed marked discrepancies. All interpretations were coded by two raters separately. Interrater agreement was assessed through the B statistic [29, 30] instead of Cohen’s Kappa because ratings of inconsistency were far less frequent than ratings of consistency. The B statistic quantifies the agreement between two raters, accounting for the agreement that would occur due to chance [29].

To address the third assumption – concerning the correspondence between daily symptom reports and diagnostic measures assessed in a clinical interview – we examined the correlation between mean diary ratings and mean scores on distinct psychopathological domains (assessed by the diagnostic interview at post assessment). Because the data did not fulfill parametric assumptions, Spearman correlation coefficients were computed. We then determined correspondence by evaluating which diary items correlated most strongly with each domain.

## Results

### Sample characteristics

Of the 443 members of the TRAILS-CC cohort who were invited to participate, 142 (32%) expressed their interest in TRAILS TRANS-ID. Of these individuals, 134 (94, 30% of the invited number of participants) enrolled in the study (76 males (57%); mean age 22.6 years old, std. 0.6). At the fourth measurement wave of TRAILS-CC, when participants were approximately 21 years old, employment and education status were assessed. The majority of participants (*N* = 73, 54%) were currently employed, of whom some with current education (*N* = 46) and some without current education (*N* = 27). Other participants either were employed in the past (*N* = 37 (28%), of whom 23 with and 14 without current education), had never been employed (*N* = 14 (10%), of whom 11 with and 3 without current education), or did not report employment or education (*N* = 10, 7%). At the first measurement wave of TRAILS-CC, when participants were approximately 11 years old, the average deviation quotient (which approximates the intelligence quotient) as measured by the Wechsler Intelligence Scale for Children [31] was 100.3 (std. 14.9). Participants’ socio-economic status, also assessed at the first measurement wave of TRAILS-CC, was based on parental education, parental employment, and household income. The participants in the present study had a socio-economic status similar to that in the general Dutch population (low: *N* = 28, 21%; medium: *N* = 69, 52%; high: *N* = 37, 28%). In terms of social functioning, 82 participants (67%) improved (baseline score < post assessment score) and 40 participants (33%) worsened (Table 1). Both at baseline and at the post-assessment, approximately one in four participants met the DSM-5 criteria for a psychiatric disorder. Of the 37 participants (28%) with a diagnosis at baseline, 26 (19%) met criteria for a single diagnosis, 7 (5%) met criteria of two diagnoses, and 4 (3%) had three or more diagnoses. Fourteen participants (10%) who initially received a diagnosis did not meet diagnostic criteria half a year later. A similar number of participants received a diagnosis at the post-assessment, but not at baseline (*N* = 12, 10%). Mean scores and standard deviations for each mini-SCAN domain and diary item are listed in supplementary tables (Table S2 and S3, Additional file 1).

**Table 1:** Sample characteristics

	Baseline (pre-diary)			Post- assessment		
	***N*** = 134			***N*** = 122		
*Life events (mean, std.)*						
No. of negative life events	1.32 (1.41)			1.07 (1.10)		
No. of positive life events	2.04 (1.18)			2.17 (1.32)		
*GVSG impairments (N, %)*^a^	No	Mild	Severe	No	Mild	Severe
Parents	116 (87%)	10 (7%)	7 (5%)	95 (78%)	15 (12%)	12 (10%)
Partner	54 (40%)	15 (11%)	10 (7%)	42 (34%)	12 (10%)	13 (11%)
Children	9 (7%)	1 (1%)	0 (0%)	7 (6%)	2 (2%)	1 (1%)
Friends	96 (72%)	21 (16%)	15 (11%)	72 (59%)	36 (30%)	10 (8%)
Education	30 (22%)	20 (15%)	5 (4%)	31 (25%)	19 (16%)	4 (3%)
Occupation	57 (43%)	26 (19%)	14 (10%)	41 (34%)	38 (31%)	23 (19%)
Household	61 (46%)	54 (40%)	14 (10%)	52 (43%)	49 (40%)	18 (15%)
Spare time	63 (47%)	31 (23%)	40 (30%)	52 (43%)	34 (28%)	36 (30%)
*mini-SCAN (N, %)*						
Anxiety disorder	14 (10%)			9 (7%)		
Mood disorder	27 (20%)			23 (19%)		
Psychotic disorder	2 (1%)			5 (4%)		
Attention deficit and/or hyperactivity disorder	8 (6%)			8 (7%)		
Substance use disorder	3 (2%)			2 (2%)		
Adjustment disorder	1 (1%)					

*Abbreviations*: *GVSG* Groningse Vragenlijst Sociaal Gedrag, a measure of social functioning in eight domains, *mini-SCAN* short version of the Schedules for Clinical Assessment in Neuropsychiatry; *std*. standard deviation

^a^Not all domains were applicable to each participant (e.g. most participants did not have children). Hence, percentages do not necessarily add up to 100%. Severity of impairments was scored based on lower and upper cut-off scores denoted in [26]

### Feasibility of the present design

**Fig. 1:**
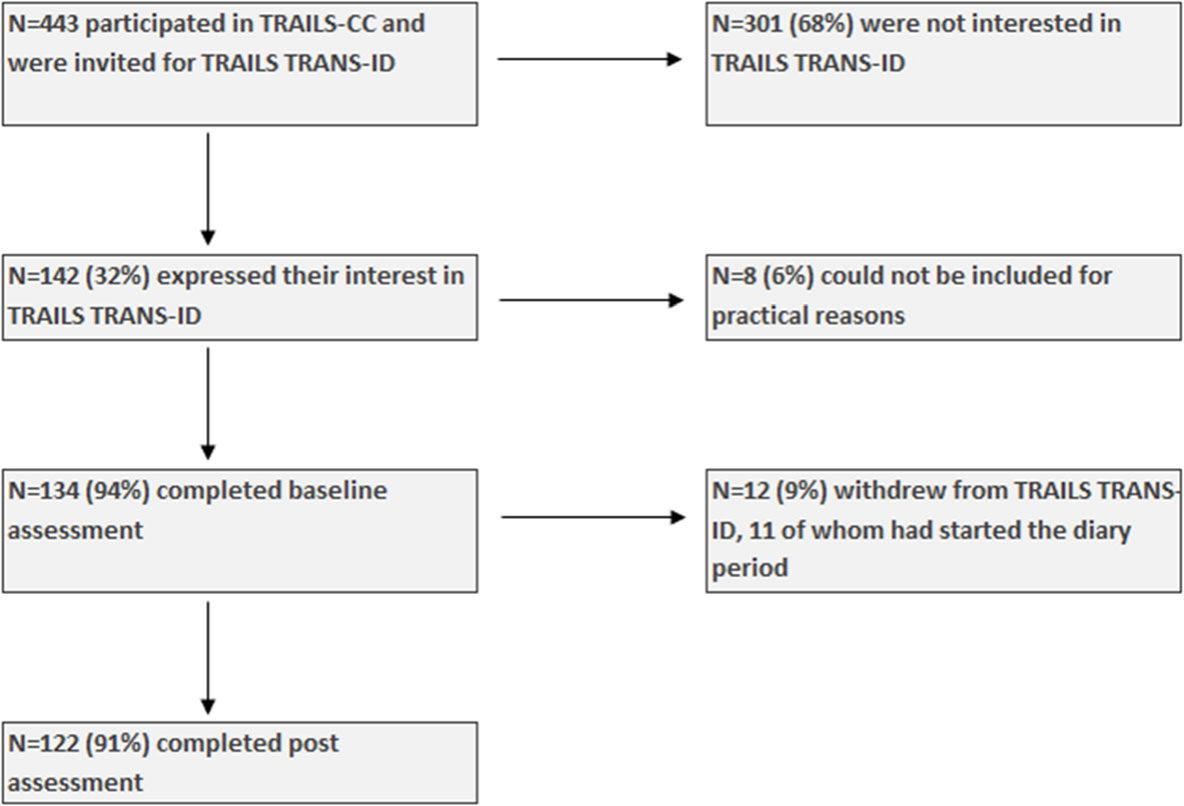
Flowchart of recruitment and inclusion procedure for TRAILS TRANS-ID. Percentages use the participants in the cell above as reference (e.g. 91% of those individuals who completed baseline assessments also completed post assessments). Practical reasons for not including participants were, for instance, long-term stay in remote areas with limited internet access

Of the 134 included participants, one decided to quit the study immediately after the baseline interview and 11 participants (9%) dropped out during the diary period, resulting in 122 participants (91% from those included) who remained in the study (Fig. 1). The 11 participants who did not complete the study remained on average 83.55 days (std. = 39.44, percentage = 46%, range 24–147) and completed 39.91 diaries (std. = 23.22, percentage = 46%, range = 9–76). They were similar to those who remained in the study with respect to age (mean 22.46 vs. 22.59 years old, t (116) = 0.69, *P* = 0.49), sex (58% vs. 57% male, χ^2^(1) < 0.01, *P* > 0.99), socio-economic status (mean standardized score 0.13 vs. 0, t (132) = 0.59, *P* = 0.56), IQ (t (132) = 1.51, *P* = 0.13), and diagnostic status at TRAILS TRANS-ID baseline (25% vs. 28% with diagnosis, odds ratio = 0.86, 95% CI = 0.14–3.73, P > 0.99). As denoted in Table 2, participants completed on average 88.5% of the diaries, and did not experience the diary period as very burdensome (mean burden: 3.21, on a scale between 1 (not at all burdensome) to 10 (extremely burdensome)). Missing a diary occurred most often because of interfering activities, such as social events, which participants were unwilling or unable to interrupt.

**Table 2:** Completion and evaluation of diary protocol

	Mean or frequency	Percentage
No. of completed diaries (std.)	162.57 (17.09)	88.5%
Burden on a scale from 1 (almost no burden) to 10 (extremely burdening) (std.)	3.21 (1.42)	
*Reason for missing a diary*		
I did not have my phone with me or was unaware of receiving a text message.	11	9%
Due to technical issues, I did not receive the text message or could not open the link.	9	7%
I was already asleep.	10	8%
I could not motivate myself to fill in in the diary.	13	11%
I was engaged in an activity I did not want to interrupt.	64	52%
I was in a location where it was impossible to fill in the diary.	7	6%
Other^a^	6	5%
Missing or not applicable^b^	2	2%

*Std*. standard deviation

^a^Other reasons for missing a diary, for instance, a broken smartphone

^b^Not applicable because participant did not miss any diaries

### Assumption 1. Variability in symptom expression

The value of intensive longitudinal designs lies in their ability to capture both between- and within-individual variability in symptom reports. These are described in Table S3 (Additional file 1). In general, items with lower mean ratings (averaged within, and subsequently across individuals) were less variable both between and within individuals. This is indicative of floor effects, for instance illustrated by the difference in mean and variability between a rarely endorsed item *44. I had a fight* (mean 7.50, std._within_ = 7.34) and a commonly endorsed item *35. I felt tired* (mean 35.06, std._within_ = 18.78). Other items where floor effects seem present are *53. I used (soft) drugs today* (mean 3.11, std._within_ = 3.57) and *32. I was easily startled* (mean 9.67, std._within_ = 7.26). In contrast, *22. I could not bring myself to do anything* and *37. It bothered me that things did not go as expected* showed high variability both between and within individuals and might thus be well-suited for intensive longitudinal designs (mean 18.40 and 21.76, std._between_ = 11.49 and 14.12, std._within_ = 15.36 and 15.95, respectively). Compared to negatively valenced items, positively valenced items generally showed higher means (range 46.88–61.41 vs. 3.11–35.06) and variability (range_between_ 13.37–16.81 vs. 5.84–17.50; range_within_ 12.79–18.34 vs. 3.57–18.79), and were hence less susceptible to floor effects. Across items, between- and within-individual variability were similarly large (overall std._between_ 13.22, overall std._within_ 13.21).

### Assumption 2. Consistent interpretations of diary items over time

The majority of interpretations that could be evaluated was rated as consistent at baseline and post assessment by both raters (460, or 95%). Other interpretations were scored as inconsistent by one rater (15, or 3%) or by both raters (7, or 1%). Hence, fluctuations in symptoms as assessed by the diary could not be attributed to changing interpretations over time (B = 0.96, indicating excellent agreement). Due to attrition and technical issues, respectively, complete data on the interpretation of the diary items (i.e. reports at both baseline and post assessment) were available for 55 of the 60 individuals who participated in the qualitative assessment. Because each participant was asked to describe ten items both at baseline and at post assessment, there were 550 sets of interpretations. Of these interpretations, 68 (12%) could not be rated due to insufficient information either at baseline or at post assessment. For instance, in some cases participants did not understand the item or could not come up with an interpretation other than the item (e.g. “Feeling down means that I feel down”).

### Assumption 3. Correspondence of diary items and clinical interview

**Fig. 2:**
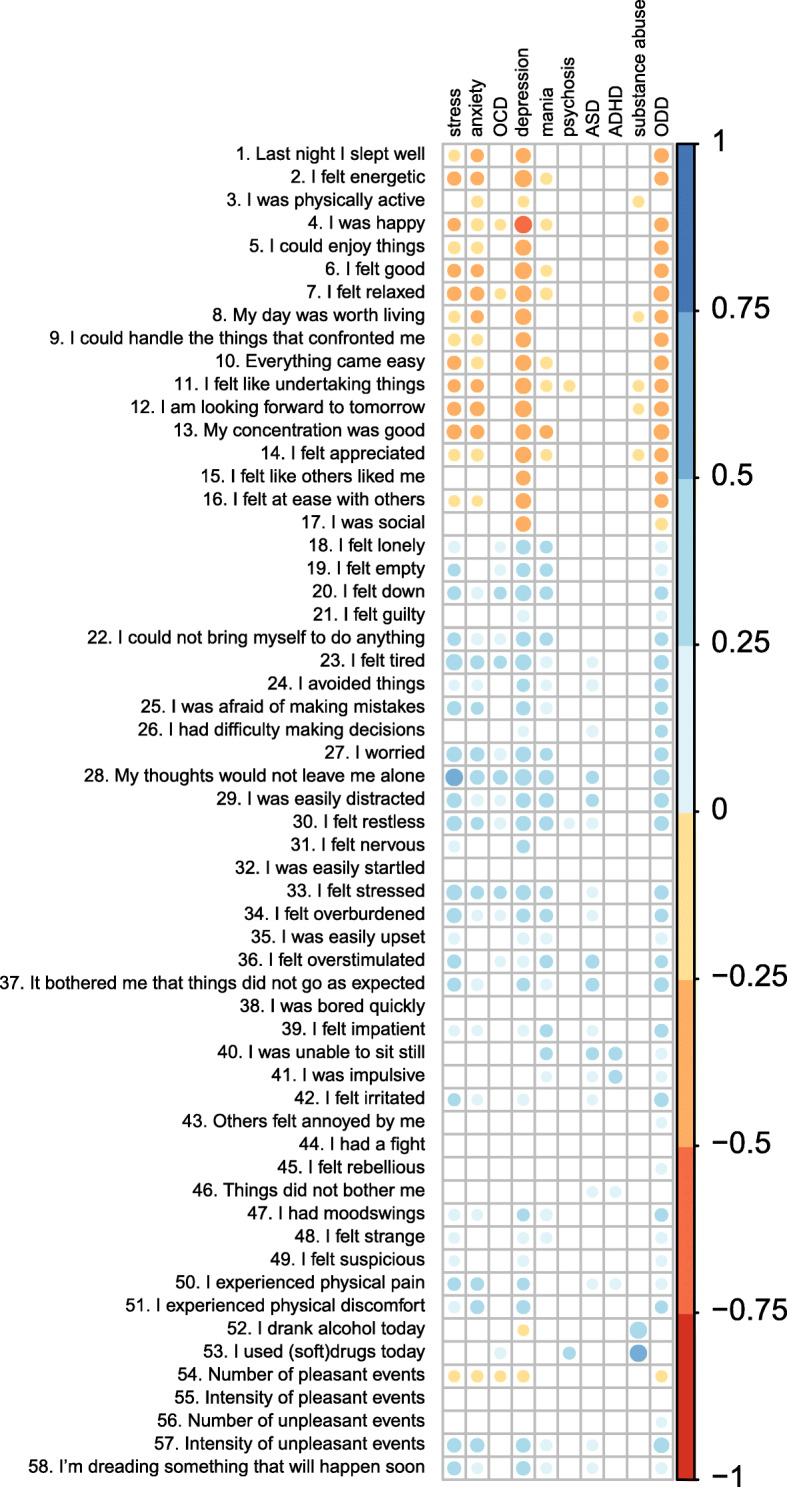
Spearman correlation coefficient between diary items and mini-SCAN domains. The color and size of the dots reflect the direction and magnitude of the correlation. Correlations that were not statistically significant (*P* > .05) were omitted from the figure

Figure 2 shows the Spearman correlations between diary item scores (within-individual means) and mini-SCAN domains scores at post assessment (see Table S5 in Additional file 1 for raw data). Overall, different diagnostic domains appeared to be related to distinct patterns in daily symptom reports (see Table S6 in Additional file 1). Internalizing problems – including the domains stress, anxiety, OCD, and depression – were strongly related to *feeling tired* (item 23), *stressed* (item 33), and *worried* (item 27). Anxiety was further covered by *experiencing physical discomfort* (item 51), while OCD and depression were more related to *feeling down* (item 20). Mania and psychosis were both related to *feeling restless* (item 30). Mania was additionally related to thought problems – reflected by *29. I was easily distracted* and *34. I felt overburdened* – while psychosis was more related to *experiencing physical discomfort* (item 51) and *using (soft) drugs* (item 53). ASD, ADHD, and substance abuse were all associated with items that specifically tapped into these domains. Specifically, ASD was primarily covered by *feeling overstimulated* (item 36) and *being bothered because things did not go as expected* (item 37), while ADHD was related to *being impulsive* (item 41) and *being unable to sit still* (item 40). The items that most strongly related to substance abuse were *using (soft) drugs* (item 53) and *drinking alcohol* (item 52). Finally, ODD was most strongly associated with a mix of items that covered internalizing domains – such as *feeling down* (item 20) – and more ODD specific items such as *feeling irritated* (item 42) and *I had mood swings (*item 47). In conclusion, daily reports of (subthreshold) symptoms aligned with retrospectively reported symptoms assessed in a diagnostic interview, and showed both specific and non-specific associations with the interview domains.

## Discussion

In the current paper, we addressed the feasibility of studying the development of psychopathological symptoms by means of six months’ intensive longitudinal measurements and evaluated three assumptions that accompany similar designs using data from the TRAILS TRANS-ID study. We found that conducting a six-month diary study in young adults at risk for mental ill-health is feasible, demonstrated by excellent compliance, low perceived burden, and low attrition. Further, all diary items showed variability over time, but some more so than others. Currently, there are no objective criteria that determine what constitutes sufficient variability. Therefore, for now, these findings indicate that differences between items exist and that researchers need to consider this when compiling the diary instrument for their intensive longitudinal design. Importantly, for the vast majority of individuals, the meaning of diary items remained consistent over time, implying that daily diaries can validly capture changes in symptoms. The correspondence between retrospective diagnostic interviews and diary reports showed how psychopathological disorders manifest in daily life. In this high-risk, transdiagnostic sample, we found that diary items differ in the number and type of psychopathological domains they cover. Taken together, our findings show that day to day monitoring of psychopathological symptoms in at risk young adults is feasible and that our items are suitable for studying symptom development. In addition, our findings may inform future intensive longitudinal studies with respect to methodological considerations (e.g. concerning the items that are most appropriate for monitoring particular psychopathological domains).

### Six-month diary studies are feasible

We showed that relatively long intensive longitudinal designs with a large sample size are feasible. To date, the majority of personalized designs in psychiatry adopted high-frequency designs for short periods of time ([16, 32–37], but see [11]). By favoring sampling duration over sampling frequency, diary studies such as TRAILS TRANS-ID capture changes that occur over a larger timescale [32]. This trade-off did not undermine the compliance of participants. Participants completed on average 89% of diaries - which is comparable to earlier studies that employed ambulatory assessments for shorter time intervals [38, 39]. Several factors might have contributed to this [40]. First, our participants have participated in scientific research for multiple years (i.e. in TRAILS-CC), which likely contributed to their commitment to also participate in this add-on study. Second, we aimed to minimize the burden introduced by the diary protocol by tailoring the protocol to participants' daily lives (e.g. by allowing participants to complete the diary on their mobile phone at the end of the day at a time convenient for them). Third, we contacted participants at least every month during the diary period which may have aided in their adherence to the study protocol [41]. Regular contact also allowed us to remove barriers that prevented participants from completing the diaries, for instance by optimizing the time at which the diary could be completed. Finally, participants were compensated for their efforts both personally and financially. The personal incentive consisted of a report on participants’ own data, which was generally received with great interest. Participants further received a financial compensation that was contingent on the number of completed diaries. In conclusion, TRAILS TRANS-ID shows that the benefits of intensive longitudinal diary studies (i.e. rich data pertaining to within-individual processes for a relatively long period of time) certainly outweigh their costs (i.e. reported burden and attrition of participants, and time and financial investment of researchers).

### Daily diaries fulfill prerequisites to measure psychopathology in daily life

Intensive longitudinal designs target within-individual changes. Therefore, the quality of measurement instruments should be evaluated on a within-individual level. Traditional longitudinal designs have addressed this, for instance, through structural equation modeling or dynamic factor modeling [42, 43]. These techniques are less suitable for IL designs because of differences in the data structure (e.g. many more assessments per individual, missing and unbalanced data, non-stationary data). The present study suggests that our instruments are fit for their purposes. Specifically, daily diaries capture meaningful variability in symptoms, in the sense that this variability cannot be attributed to changes in the (subjectively perceived) content of items and reasonably corresponds to variability in diagnostic measures. First, a qualitative analysis of individuals’ interpretation of diary items immediately before and after the six-month diary period showed that participants’ interpretations were highly consistent over time. Participants were, however, sometimes unable to understand or verbalize certain items. This highlights the importance of verifying the comprehensibility of self-report measures, particularly in populations with average to limited verbal or introspective abilities [44]. Pilot studies in samples with a background similar to that of the target population – such as the pilot study conducted prior to the current project – aid in accomplishing appropriate phrasing of items. Second, evaluation of the correspondence between diary items and diagnostic interviews showed that retrospectively assessed diagnoses reasonably manifested in daily symptom reports (e.g. ADHD and being unable to sit still). This shows that intensive longitudinal data and cross-sectional data might enrich each other by each providing partly overlapping and partly unique information concerning individuals’ mental health. Together, these sources of information can provide a detailed picture of how, when, and for whom psychopathological symptoms develop.

### Methodological issues

First, IL designs require a careful consideration of the time scale at which processes unfold. While mood changes within minutes or hours, symptoms may evolve over longer timescales [45]. This means that assessing dynamics in mood versus symptoms requires different designs. The within-individual variability in diary ratings found in the current study illustrates that the dynamics of psychopathological symptoms – or subthreshold expressions thereof – can indeed be captured by daily assessments. Second, it is possible that the individuals included in studies using IL designs are not representative of the general population, for instance because individuals who are willing to monitor their mental state for several consecutive weeks or months might be relatively more motivated [46]. However, in the current study, financial compensation was given to participants which may have limited this issue. Further research is needed to explore who are most likely to enroll in and complete IL studies. Finally, the repeated assessments adopted in IL designs might evoke reactivity [41]. That is, repeatedly assessing one’s mental state might affect one’s (appraisal of) experiences, thoughts, and feelings. Reactivity might be limited by providing participants with their own reports *after* instead of *during* the diary study – as was done in the current study. This is because it is less likely that participants alter their responses based on earlier responses when they are not confronted with earlier responses. In conclusion, designing and evaluating studies using intensive longitudinal methods requires appreciation of the timescale of interest as well as participants’ responses to these methods.

### Future implications

The established feasibility of intensive longitudinal diary studies call for a brief overview of the types of theories that could be investigated using these designs. Network theory [47, 48] and complex systems theory [1, 49, 50], for instance, both propose that the dynamics of symptoms – i.e. the temporal variation in symptoms within individuals – might be predictive of long-term prognosis. According to network theory, psychopathology can be conceptualized as a network of (causally) interacting symptoms. The more symptoms trigger each other, the more clusters of co-occurring symptoms arise. The strength and the structure of associations between symptoms may thus reveal individuals’ vulnerability towards developing psychopathology. Second, psychopathology has been proposed to behave according to principles common to complex systems. This means that indicators of instability, referred to as early warning signals (EWS), may be informative of sudden transitions in symptom severity [2, 51, 52]. These early warning signals are based on the temporal dynamics of symptoms, for instance reflected in the degree to which they carry over from one moment to the next. Because TRAILS TRANS-ID includes a broad set of symptoms spanning multiple disorder domains, these data may also be used to investigate whether EWS are informative of the type of symptom shifts in addition to shifts in symptom severity [49]. Both network and complex systems approaches to psychopathology raise hypotheses on the level of individuals. That is, changes in symptoms within an individual – operationalized either as network characteristics or as early warning signals – may be informative of the future development of that individual. Due to the individual-centered approach, the conclusions that follow from these hypotheses could directly translate to clinical practice. In order to examine the clinical utility of network and complex systems approaches, personalized designs such as TRAILS TRANS-ID are needed.

In addition to deriving individual-specific models, intensive longitudinal data collected in comparatively large samples such as in the TRAILS TRANS-ID study also allow for investigating what temporal processes may be shared by multiple individuals. Although some have argued that group-findings based on IL data do not translate well to individuals [53], it is also unlikely that every individual is unique and requires their own model describing psychological processes. Newly developed clustering techniques such as Group Iterative Multiple Model Estimation (GIMME [54]) can be applied to IL data to detect (sub) groups of individuals that share temporal associations. Its embedding in the larger TRAILS cohort study yields the TRAILS-TRANS-ID study the unique possibility to investigate associations between current day to day symptom patterns to previously collected developmental and contextual variables as well as future outcomes.

## Conclusion

TRAILS TRANS-ID has shown that prospectively monitoring symptoms as they develop over time in at risk young adults is within reach. We have demonstrated feasibility, within-person variability, consistency in meaning and correspondence with diagnostic outcomes. It is our hope that this pioneering study provides the groundwork for intensive longitudinal designs over extended periods that aim to investigate how psychopathology unfolds over time within individuals. The outcomes of such intensive longitudinal designs allow for investigating individual-level hypotheses derived from novel conceptualizations of psychopathology such as network theory and complex systems theory.

## Supplementary information

**Additional file 1: Table S1.** Financial compensation for the diary period. **Fig. S1.** Outline of the TRAILS TRANS-ID study design. At baseline and post assessment, the following instruments were employed: diagnostic interview (short version of the Schedules for Clinical Assessment in Neuropsychiatry, 23), aggressive behavior subscale of the Adult Self Report (24), Groningse Vragenlijst Sociaal Gedrag (17), List of Threatening Experiences (26). The same instruments will be administered during the follow-up assessment, which was not completed yet at the time of submission. For a subset of 60 participants, the baseline and post assessments were complemented by a qualitative assessment that served to establish the validity of diary items. **Table S2.** Scores per mini-SCAN domain. **Table S3.** Descriptives diary items. **Table S4.** Scoring of the mini-SCAN interview. **Table S5.** Correlation between mean ratings on diary items and mini-SCAN domains assessed during the post assessment interview. **Table S6.** Five diary items that correlated most strongly with each psychopathological domain assessed by the mini-SCAN (left: highest Spearman correlation coefficient).

## Data Availability

The datasets analysed during the current study are not publicly available due to the possibility to identify participants based on their clinical and diary data (European law).

## Abbreviations

ADHD: Attention Deficit Hyperactivity Disorder
ASD: Autism spectrum disorder
ASR: Adult Self Report
EWS: Early warning signals
GVSG: Groningen Social Behaviour Questionnaire (in Dutch: Groningse Vragenlijst Sociaal Gedrag)
IL: Intensive longitudinal
Mini-SCAN: short version of the Schedules for Clinical Assessment in Neuropsychiatry
OCD: Obsessive-compulsive disorder
ODD: Oppositional defiant disorder
PTSD: Post-traumatic stress disorder
Std: Standard deviation
TRAILS: TRacking Adolescents’ Individual Lives Survey
TRANS-ID: Transitions In Depression

## Footnotes

1 Unlike TRANS-ID, TRAILS TRANS-ID does not solely focus on depression, but aims to examine psychopathology across diagnostic borders, including, but not limited to, mood disorders, anxiety disorders, subthreshold psychosis, thought problems, Attention Deficit Hyperactivity Disorder, Autism Spectrum Disorder and aggression.
