# Supplementary material for: Measuring psychopathology as it unfolds in daily life: addressing key assumptions of intensive longitudinal methods in the TRAILS TRANS-ID study
Source: BMC Psychiatry. 2020 Jul 6;20:351. doi: 10.1186/s12888-020-02674-1 (PMC7336426; doi:10.1186/s12888-020-02674-1)
Supplement: Supplementary file 1 — Additional file 1: Table S1. Financial compensation for the diary period. Fig. S1. Outline of the TRAILS TRANS-ID study design. At baseline and post assessment, the following instruments were employed: diagnostic interview (short version of the Schedules for Clinical Assessment in Neuropsychiatry, 23), aggressive behavior subscale of the Adult Self Report (24), Groningse Vragenlijst Sociaal Gedrag (17), List of Threatening Experiences (26). The same instruments will be administered during the follow-up assessment, which was not completed yet at the time of submission. For a subset of 60 participants, the baseline and post assessments were complemented by a qualitative assessment that served to establish the validity of diary items. Table S2. Scores per mini-SCAN domain. Table S3. Descriptives diary items. Table S4. Scoring of the mini-SCAN interview. Table S5. Correlation between mean ratings on diary items and mini-SCAN domains assessed during the post assessment interview. Table S6. Five diary items that correlated most strongly with each psychopathological domain assessed by the mini-SCAN (left: highest Spearman correlation coefficient). [file 12888_2020_2674_MOESM1_ESM.docx]

**Additional file 1**

**Financial compensation**

Participants received €20,- per interview (baseline, post assessment, and follow-up). Additionally, participants received compensation for the completion of daily diaries, which was contingent on the amount of diaries completed (see table below). The maximum compensation amounted to €200,- (*i.e.* €60,- for participating in all three clinical interviews, together with €140,- for completing ≥90% of the diaries).

**Table S1.** Financial compensation for the diary period

| Amount of daily diaries completed | Financial compensation |
| --- | --- |
| <128 (<70%) | €40,- |
| 128-145 (70-79%) | €80,- |
| 146-163 (80-89%) | €130,- |
| ≥164 (≥90%) | €140,- |

Daily diary: symptom assessments (6 months)

Day 1

Day 2, Day 3 ….. Day 183

baseline assessment

post assessment

follow-up

assessment

±12 months

**Fig. S1.** Outline of the TRAILS TRANS-ID study design. At baseline and post assessment, the following instruments were employed: diagnostic interview (short version of the Schedules for Clinical Assessment in Neuropsychiatry, 23), aggressive behavior subscale of the Adult Self Report (24), Groningse Vragenlijst Sociaal Gedrag (17), List of Threatening Experiences (26). The same instruments will be administered during the follow-up assessment, which was not completed yet at the time of submission. For a subset of 60 participants, the baseline and post assessments were complemented by a qualitative assessment that served to establish the validity of diary items.

**Pilot study**

Participants for the pilot study were recruited in a local school for intermediate vocational education. Adolescents were informed about the purposes of the pilot study and provided written informed consent. In a semi-structured interview, individuals were asked to report their intuitive interpretation of diary items and to rate the extent to which this item described their current mood. Individuals were explicitly instructed to provide their personal view on items and were ensured that answers were not labeled ‘wrong’ or ‘right’. After completion of the interview, individuals received a voucher worth €20,-.

Interpretations were noted and discussed among the research team. Of the 63 items assessed in the pilot study, nine items were rephrased in order to better match participants’ vocabulary. Five items were deleted due to substantial perceived overlap with other items or due to frequently occurring misunderstandings. Finally, one item was added, resulting in a total of 59 items concerning mood (*e.g. I felt happy*), (subthreshold) psychopathological symptoms (*e.g. I felt stressed*), somatic experiences (*e.g. I felt ill*), sleep quality (*e.g. I slept well*), and event appraisal (*e.g. pleasantness of the most important event during the previous day*).

**Table S2.** Scores per mini-SCAN domain

| **Domain** | **Baseline** | **Range** | **Post assessment** | **Range** |
| --- | --- | --- | --- | --- |
| Stress | 0.51 (0.48) | 0-2 | 0.57 (0.55) | 0-1.83 |
| Anxiety | 0.13 (0.23) | 0-1.69 | 0.12 (0.20) | 0-1.19 |
| OCD | 0.18 (0.36) | 0-1.50 | 0.16 (0.33) | 0-1.56 |
| Depression | 0.32 (0.35) | 0-1.50 | 0.39 (0.41) | 0-1.64 |
| Mania | 0.13 (0.24) | 0-1.78 | 0.16 (0.25) | 0-1.78 |
| Psychosis | 0.02 (0.07) | 0-0.55 | 0.02 (0.08) | 0-0.50 |
| ASD | 0.43 (0.39) | 0-1.50 | 0.41 (0.40) | 0-1.70 |
| ADHD | 0.66 (0.46) | 0-1.79 | 0.67 (0.43) | 0-1.70 |
| Substance abuse | 0.16 (0.19) | 0-1.71 | 0.20 (0.19) | 0-1.29 |
| ODD | 0.35 (0.30) | 0-1.60 | 0.37 (0.32) | 0-1.40 |

Number denote mean scores per domain; numbers between brackets denote standard deviations. *Abbreviations:* OCD=obsessive-compulsive disorder; ASD=autism spectrum disorder; ADHD=attention deficit/hyperactivity disorder; ODD=oppositional defiant disorder.

**Table S3.** Descriptives diary items

| Diary item | within-individual mean | between-individual std. | within-individual std. |
| --- | --- | --- | --- |
| 1. Last night I slept well | 55.09 | 13.37 | 16.29 |
| 2. I felt energetic | 47.79 | 14.76 | 17.09 |
| 3. I was physically active | 46.88 | 15.26 | 18.03 |
| 4. I was happy | 57.71 | 15.83 | 14.23 |
| 5. I could enjoy things | 58.57 | 14.37 | 14.84 |
| 6. I felt good | 58.68 | 14.67 | 15.23 |
| 7. I felt relaxed | 50.20 | 14.93 | 17.72 |
| 8. My day was worth living | 61.41 | 14.98 | 15.07 |
| 9. I could handle the things that confronted me | 61.35 | 14.08 | 15.20 |
| 10. Everything came easy | 58.24 | 14.79 | 12.79 |
| 11. I felt like undertaking things | 48.41 | 14.98 | 18.34 |
| 12. I am looking forward to tomorrow | 55.79 | 16.58 | 16.01 |
| 13. My concentration was good | 51.87 | 14.06 | 15.95 |
| 14. I felt appreciated | 56.68 | 16.81 | 14.48 |
| 15. I felt like others liked me | 58.16 | 15.30 | 14.42 |
| 16. I felt at ease with others | 59.01 | 14.64 | 15.16 |
| 17. I was social | 57.85 | 14.56 | 14.89 |
| 18. I felt lonely | 14.63 | 13.31 | 11.24 |
| 19. I felt empty | 14.08 | 12.48 | 10.95 |
| 20. I felt down | 15.62 | 13.64 | 12.20 |
| 21. I felt guilty | 12.64 | 10.74 | 10.17 |
| 22. I could not bring myself to do anything | 18.40 | 11.49 | 15.36 |
| 23. I felt tired | 35.06 | 15.86 | 18.79 |
| 24. I avoided things | 16.00 | 12.68 | 12.47 |
| 25. I was afraid of making mistakes | 16.61 | 12.67 | 12.34 |
| 26. I had difficulty making decisions | 16.65 | 11.93 | 12.75 |
| 27. I worried | 21.60 | 14.23 | 14.61 |
| 28. My thoughts would not leave me alone | 23.68 | 17.14 | 14.45 |
| 29. I was easily distracted | 19.35 | 13.48 | 14.01 |
| 30. I felt restless | 19.55 | 13.71 | 13.66 |
| 31. I felt nervous | 15.46 | 11.75 | 12.89 |
| 32. I was easily startled | 9.67 | 9.18 | 7.26 |
| 33. I felt stressed | 18.97 | 13.87 | 13.99 |
| 34. I felt overburdened | 18.03 | 14.28 | 13.27 |
| 35. I was easily upset | 12.96 | 10.76 | 10.99 |
| 36. I felt overstimulated | 17.28 | 14.94 | 12.52 |
| 37. It bothered me that things did not go as expected | 21.76 | 14.12 | 15.95 |
| 38. I was bored quickly | 13.19 | 9.70 | 10.65 |
| 39. I felt impatient | 14.28 | 10.69 | 11.05 |
| 40. I was unable to sit still | 17.75 | 13.74 | 13.12 |
| 41. I was impulsive | 20.76 | 15.44 | 12.99 |
| 42. I felt irritated | 17.03 | 11.62 | 13.59 |
| 43. Others felt annoyed by me | 12.70 | 9.62 | 9.65 |
| 44. I had a fight | 7.50 | 8.11 | 7.34 |
| 45. I felt rebellious | 12.15 | 10.59 | 9.07 |
| 46. Things did not bother me | 21.18 | 16.01 | 13.82 |
| 47. I had moodswings | 15.59 | 12.98 | 11.67 |
| 48. I felt strange | 13.22 | 11.31 | 10.06 |
| 49. I felt suspicious | 11.74 | 11.17 | 8.49 |
| 50. I experienced physical pain | 17.51 | 14.05 | 13.88 |
| 51. I experienced physical discomfort | 14.93 | 11.09 | 13.44 |
| 52. I drank alcohol today | 7.92 | 8.07 | 10.24 |
| 53. I used (soft)drugs today | 3.11 | 5.84 | 3.57 |
| 54. Number of pleasant events | 35.64 | 17.48 | 14.47 |
| 55. Intensity of pleasant events | 46.81 | 17.85 | 17.74 |
| 56. Number of unpleasant events | 15.94 | 9.72 | 10.95 |
| 57. Intensity of unpleasant events | 23.47 | 13.75 | 17.49 |
| 58. I’m dreading something that will happen soon | 23.07 | 17.50 | 15.06 |

Within-individual mean = averaged mean item score per individual; between-individual std. = standard deviation of mean item scores; within-individual std. = averaged standard deviation of item scores per individual.

**Details on scoring for the mini-SCAN interview**

Table S4 provides an overview of the scoring rules adopted for the mini-SCAN interview. The mini-SCAN interview is typically administered to infer whether individuals meet the diagnostic criteria for a psychiatric disorder, and therefore only assesses clinically significant symptoms. For the purposes of the present study, a more dimensional rating of mental health was adopted. That is, we rated symptoms that were insufficiently severe, enduring, or frequent to qualify as a sign of psychopathology – but nevertheless present and distressing – as subthreshold (26). To infer whether individuals met the criteria for a psychiatric disorder, the original, dichotomous rating scheme was used. Hence, only symptoms coded as clinical were taken into account when inferring whether participants met the criteria for a psychiatric disorder.

**Table S4.** Scoring of the mini-SCAN interview

| Description | Mini-SCAN rating in TRAILS TRANS-ID | Original mini-SCAN rating* |
| --- | --- | --- |
| Symptom causes significant burden due to impairments in functioning, duration and/or frequency of occurrence. | Clinical | Present |
| Symptom is endorsed, but does not cause significant burden (*e.g.* because of mild severity, short duration or infrequent occurrence). | Subthreshold | Absent |
| Symptom is absent. | Absent | Absent |

*See reference (15).

| mini-SCAN domain | Diary item |  |  |  |  |
| --- | --- | --- | --- | --- | --- |
| stress | 28. My thoughts would not leave me alone | 23. I felt tired | 33. I felt stressed | 27. I worried | 30. I felt restless |
| anxiety | 28. My thoughts would not leave me alone | 27. I worried | 57. Intensity of unpleasant events | 51. I experienced physical discomfort | 23. I felt tired |
| OCD | 28. My thoughts would not leave me alone | 23. I felt tired | 33. I felt stressed | 20. I felt down | 27. I worried |
| depression | 28. My thoughts would not leave me alone | 20. I felt down | 23. I felt tired | 27. I worried | 33. I felt stressed |
| mania | 28. My thoughts would not leave me alone | 29. I was easily distracted | 30. I felt restless | 34. I felt overburdened | 20. I felt down |
| psychosis | 53. I used (soft)drugs today | 30. I felt restless | 28. My thoughts would not leave me alone | 51. I experienced physical discomfort | 57. Intensity of unpleasant events |
| ASD | 36. I felt overstimulated | 37. It bothered me that things did not go as expected | 28. My thoughts would not leave me alone | 29. I was easily distracted | 40. I was unable to sit still |
| ADHD | 41. I was impulsive | 40. I was unable to sit still | 50. I experienced physical pain | 46. Things did not bother me | 39. I felt impatient |
| substance abuse | 53. I used (soft)drugs today | 52. I drank alcohol today | 22. I could not bring myself to do anything | 28. My thoughts would not leave me alone | 20. I felt down |
| ODD | 28. My thoughts would not leave me alone | 57. Intensity of unpleasant events | 29. I was easily distracted | 30. I felt restless | 23. I felt tired |

**Table S6.** Five diary items that correlated most strongly with each psychopathological domain assessed by the mini-SCAN (left: highest Spearman correlation coefficient).

**Table S5.** Correlation between mean ratings on diary items and mini-SCAN domains assessed during the post assessment interview

|  | **Tension** | | | **Anxiety** | | | | **OCD** | | | | **Depression** | | | | **Mania** | | | | **Psychosis** | | | | **ASD** | | | | **ADHD** | | | | | **Substance** | | | | **ODD** | | | |  |  |  |  |
| --- | --- | --- | --- | --- | --- | --- | --- | --- | --- | --- | --- | --- | --- | --- | --- | --- | --- | --- | --- | --- | --- | --- | --- | --- | --- | --- | --- | --- | --- | --- | --- | --- | --- | --- | --- | --- | --- | --- | --- | --- | --- | --- | --- | --- |
|  | **r** | **P** | | **r** | | **P** | | **r** | | **P** | | **r** | | **P** | | **r** | | **P** | | **r** | | **P** | | **r** | | **P** | | **r** | | **P** | | | **r** | | **P** | | **r** | | **P** | |  |  |  |  |
| 1. Last night I slept well | -0.21 | 0.02 | | -0.27 | | <.01 | | -0.08 | | 0.41 | | -0.37 | | <.01 | | -0.17 | | 0.06 | | -0.13 | | 0.14 | | -0.06 | | 0.55 | | -0.08 | | 0.36 | | | -0.11 | | 0.21 | | -0.35 | | <.01 | |  |  |  |  |
| 2. I felt energetic | -0.32 | <.01 | | -0.29 | | <.01 | | -0.14 | | 0.11 | | -0.49 | | <.01 | | -0.21 | | 0.02 | | -0.13 | | 0.15 | | -0.11 | | 0.23 | | -0.09 | | 0.31 | | | -0.07 | | 0.46 | | -0.32 | | <.01 | |  |  |  |  |
| 3. I was physically active | -0.06 | 0.50 | | -0.21 | | 0.02 | | -0.03 | | 0.77 | | -0.20 | | 0.03 | | -0.02 | | 0.80 | | -0.11 | | 0.25 | | 0.08 | | 0.37 | | -0.02 | | 0.83 | | | -0.20 | | 0.03 | | -0.13 | | 0.16 | |  |  |  |  |
| 4. I was happy | -0.28 | <.01 | | -0.25 | | 0.01 | | -0.19 | | 0.03 | | -0.50 | | <.01 | | -0.21 | | 0.02 | | -0.11 | | 0.23 | | -0.04 | | 0.68 | | 0.02 | | 0.81 | | | -0.17 | | 0.06 | | -0.33 | | <.01 | |  |  |  |  |
| 5. I could enjoy things | -0.24 | 0.01 | | -0.23 | | 0.01 | | -0.17 | | 0.06 | | -0.42 | | <.01 | | -0.14 | | 0.11 | | -0.14 | | 0.11 | | -0.07 | | 0.48 | | <.01 | | 0.98 | | | -0.14 | | 0.13 | | -0.33 | | <.01 | |  |  |  |  |
| 6. I felt good | -0.30 | <.01 | | -0.26 | | <.01 | | -0.14 | | 0.13 | | -0.48 | | <.01 | | -0.21 | | 0.02 | | -0.10 | | 0.30 | | -0.06 | | 0.54 | | 0.01 | | 0.89 | | | -0.12 | | 0.20 | | -0.34 | | <.01 | |  |  |  |  |
| 7. I felt relaxed | -0.34 | <.01 | | -0.32 | | <.01 | | -0.19 | | 0.04 | | -0.44 | | <.01 | | -0.24 | | 0.01 | | -0.13 | | 0.14 | | -0.13 | | 0.14 | | -0.04 | | 0.69 | | | -0.13 | | 0.16 | | -0.41 | | <.01 | |  |  |  |  |
| 8. My day was worth living | -0.22 | 0.02 | | -0.26 | | <.01 | | -0.07 | | 0.43 | | -0.43 | | <.01 | | -0.14 | | 0.13 | | -0.10 | | 0.29 | | -0.02 | | 0.85 | | 0.01 | | 0.93 | | | -0.18 | | 0.05 | | -0.30 | | <.01 | |  |  |  |  |
| 9. I could handle the things that confronted me | -0.25 | 0.01 | | -0.20 | | 0.03 | | -0.14 | | 0.12 | | -0.39 | | <.01 | | -0.15 | | 0.11 | | -0.06 | | 0.51 | | -0.16 | | 0.08 | | -0.10 | | 0.29 | | | -0.15 | | 0.09 | | -0.33 | | <.01 | |  |  |  |  |
| 10. Everything came easy | -0.32 | <.01 | | -0.23 | | 0.01 | | -0.16 | | 0.08 | | -0.42 | | <.01 | | -0.24 | | 0.01 | | -0.07 | | 0.42 | | -0.12 | | 0.20 | | 0.01 | | 0.90 | | | -0.11 | | 0.25 | | -0.34 | | <.01 | |  |  |  |  |
| 11. I felt like undertaking things | -0.27 | <.01 | | -0.28 | | <.01 | | -0.11 | | 0.23 | | -0.44 | | <.01 | | -0.21 | | 0.02 | | -0.21 | | 0.02 | | 0.01 | | 0.94 | | -0.10 | | 0.28 | | | -0.20 | | 0.03 | | -0.32 | | <.01 | |  |  |  |  |
| 12. I am looking forward to tomorrow | -0.31 | <.01 | | -0.34 | | <.01 | | -0.09 | | 0.32 | | -0.46 | | <.01 | | -0.17 | | 0.06 | | -0.17 | | 0.05 | | -0.07 | | 0.47 | | -0.03 | | 0.76 | | | -0.18 | | 0.04 | | -0.35 | | <.01 | |  |  |  |  |
| 13. My concentration was good | -0.36 | <.01 | | -0.32 | | <.01 | | -0.11 | | 0.22 | | -0.41 | | <.01 | | -0.29 | | <.01 | | -0.14 | | 0.11 | | -0.15 | | 0.10 | | -0.16 | | 0.08 | | | -0.16 | | 0.08 | | -0.40 | | <.01 | |  |  |  |  |
| 14. I felt appreciated | -0.22 | 0.01 | | -0.25 | | 0.01 | | -0.12 | | 0.19 | | -0.42 | | <.01 | | -0.21 | | 0.02 | | -0.16 | | 0.07 | | -0.06 | | 0.49 | | 0.03 | | 0.72 | | | -0.20 | | 0.03 | | -0.32 | | <.01 | |  |  |  |  |
| 15. I felt like others liked me | -0.16 | 0.07 | | -0.16 | | 0.08 | | -0.12 | | 0.20 | | -0.35 | | <.01 | | -0.14 | | 0.13 | | -0.10 | | 0.25 | | -0.06 | | 0.48 | | 0.03 | | 0.70 | | | -0.17 | | 0.06 | | -0.26 | | <.01 | |  |  |  |  |
| 16. I felt at ease with others | -0.20 | 0.03 | | -0.18 | | 0.04 | | -0.16 | | 0.08 | | -0.40 | | <.01 | | -0.17 | | 0.06 | | -0.12 | | 0.17 | | -0.11 | | 0.22 | | -0.05 | | 0.55 | | | -0.18 | | 0.05 | | -0.30 | | <.01 | |  |  |  |  |
| 17. I was social | -0.14 | 0.11 | | -0.14 | | 0.12 | | -0.16 | | 0.07 | | -0.39 | | <.01 | | -0.13 | | 0.15 | | -0.14 | | 0.13 | | -0.09 | | 0.31 | | 0.02 | | 0.79 | | | -0.15 | | 0.11 | | -0.25 | | 0.01 | |  |  |  |  |
| 18. I felt lonely | 0.24 | 0.01 | | 0.16 | | 0.08 | | 0.18 | | 0.05 | | 0.35 | | <.01 | | 0.25 | | 0.01 | | 0.07 | | 0.41 | | 0.07 | | 0.44 | | -0.03 | | 0.74 | | | 0.10 | | 0.26 | | 0.24 | | 0.01 | |  |  |  |  |
| 19. I felt empty | 0.26 | <.01 | | 0.15 | | 0.10 | | 0.21 | | 0.02 | | 0.32 | | <.01 | | 0.26 | | <.01 | | 0.10 | | 0.30 | | 0.06 | | 0.53 | | -0.02 | | 0.81 | | | 0.07 | | 0.43 | | 0.24 | | 0.01 | | | |  |  |
| 20. I felt down | 0.30 | <.01 | | 0.23 | | 0.01 | | 0.25 | | <.01 | | 0.42 | | <.01 | | 0.28 | | <.01 | | 0.09 | | 0.33 | | 0.08 | | 0.40 | | -0.03 | | 0.78 | | | 0.11 | | 0.24 | | 0.28 | | <.01 | | | |  |  |
| 21. I felt guilty | 0.18 | 0.05 | | 0.14 | | 0.14 | | 0.10 | | 0.26 | | 0.24 | | 0.01 | | 0.18 | | 0.05 | | 0.01 | | 0.92 | | 0.08 | | 0.37 | | -0.04 | | 0.64 | | | <.01 | | 0.97 | | 0.19 | | 0.04 | | | |  |  |
| 22. I could not bring myself to do anything | 0.29 | <.01 | | 0.24 | | 0.01 | | 0.21 | | 0.02 | | 0.35 | | <.01 | | 0.28 | | <.01 | | 0.12 | | 0.20 | | 0.16 | | 0.09 | | 0.10 | | 0.29 | | | 0.16 | | 0.08 | | 0.29 | | <.01 | | | |  |  |
| 23. I felt tired | 0.45 | <.01 | | 0.31 | | <.01 | | 0.27 | | <.01 | | 0.42 | | <.01 | | 0.23 | | 0.01 | | 0.08 | | 0.36 | | 0.20 | | 0.03 | | 0.02 | | 0.86 | | | -0.09 | | 0.35 | | 0.35 | | <.01 | | | |  |  |
| 24. I avoided things | 0.18 | 0.04 | | 0.19 | | 0.03 | | 0.11 | | 0.23 | | 0.27 | | <.01 | | 0.19 | | 0.04 | | 0.12 | | 0.18 | | 0.25 | | 0.01 | | 0.14 | | 0.13 | | | 0.04 | | 0.64 | | 0.30 | | <.01 | | | |  |  |
| 25. I was afraid of making mistakes | 0.32 | <.01 | | 0.25 | | 0.01 | | 0.17 | | 0.06 | | 0.32 | | <.01 | | 0.23 | | 0.01 | | 0.01 | | 0.89 | | 0.16 | | 0.08 | | -0.02 | | 0.79 | | | -0.01 | | 0.87 | | 0.27 | | <.01 | | | |  |  |
| 26. I had difficulty making decisions | 0.17 | 0.06 | | 0.14 | | 0.12 | | 0.13 | | 0.15 | | 0.18 | | 0.05 | | 0.18 | | 0.05 | | 0.08 | | 0.41 | | 0.23 | | 0.01 | | 0.08 | | 0.39 | | | 0.06 | | 0.51 | | 0.25 | | <.01 | | | |  |  |
| 27. I worried | 0.40 | <.01 | | 0.33 | | <.01 | | 0.25 | | 0.01 | | 0.40 | | <.01 | | 0.25 | | <.01 | | 0.08 | | 0.40 | | 0.12 | | 0.20 | | -0.04 | | 0.64 | | | 0.10 | | 0.28 | | 0.31 | | <.01 | | | |  |  |
| 28. My thoughts would not leave me alone | 0.51 | <.01 | | 0.35 | | <.01 | | 0.35 | | <.01 | | 0.46 | | <.01 | | 0.37 | | <.01 | | 0.16 | | 0.07 | | 0.26 | | <.01 | | 0.05 | | 0.60 | | | 0.12 | | 0.19 | | 0.42 | | <.01 | | | |  |  |
| 29. I was easily distracted | 0.37 | <.01 | | 0.24 | | 0.01 | | 0.20 | | 0.03 | | 0.37 | | <.01 | | 0.35 | | <.01 | | 0.10 | | 0.28 | | 0.26 | | <.01 | | 0.10 | | 0.26 | | | 0.06 | | 0.49 | | 0.35 | | <.01 | | | |  |  |
| 30. I felt restless | 0.38 | <.01 | | 0.26 | | <.01 | | 0.23 | | 0.01 | | 0.37 | | <.01 | | 0.33 | | <.01 | | 0.18 | | 0.05 | | 0.23 | | 0.01 | | 0.05 | | 0.62 | | | 0.07 | | 0.42 | | 0.35 | | <.01 | | | |  |  |
| 31. I felt nervous | 0.22 | 0.01 | | 0.17 | | 0.06 | | 0.14 | | 0.13 | | 0.28 | | <.01 | | 0.18 | | 0.05 | | 0.05 | | 0.59 | | 0.10 | | 0.26 | | -0.06 | | 0.52 | | | 0.03 | | 0.75 | | 0.17 | | 0.06 | | | |  |  |
| 32. I was easily startled | 0.06 | 0.49 | | 0.09 | | 0.32 | | 0.05 | | 0.57 | | 0.12 | | 0.20 | | 0.09 | | 0.33 | | -0.06 | | 0.50 | | 0.05 | | 0.57 | | -0.10 | | 0.30 | | | 0.03 | | 0.76 | | 0.09 | | 0.33 | | | |  |  |
| 33. I felt stressed | 0.40 | <.01 | | 0.28 | | <.01 | | 0.26 | | <.01 | | 0.38 | | <.01 | | 0.28 | | <.01 | | 0.07 | | 0.44 | | 0.20 | | 0.03 | | <.01 | | 0.96 | | | 0.04 | | 0.66 | | 0.33 | | <.01 | | | |  |  |
| 34. I felt overburdened | 0.37 | <.01 | | 0.20 | | 0.02 | | 0.20 | | 0.03 | | 0.31 | | <.01 | | 0.28 | | <.01 | | 0.01 | | 0.94 | | 0.20 | | 0.03 | | 0.10 | | 0.30 | | | -0.03 | | 0.78 | | 0.29 | | <.01 | | | |  |  |
| 35. I was easily upset | 0.23 | 0.01 | | 0.16 | | 0.08 | | 0.14 | | 0.11 | | 0.25 | | 0.01 | | 0.22 | | 0.01 | | -0.01 | | 0.93 | | 0.13 | | 0.17 | | <.01 | | 1.00 | | | 0.04 | | 0.63 | | 0.24 | | 0.01 | | | |  |  |
| 36. I felt overstimulated | 0.31 | <.01 | | 0.15 | | 0.10 | | 0.19 | | 0.04 | | 0.23 | | 0.01 | | 0.28 | | <.01 | | 0.03 | | 0.75 | | 0.30 | | <.01 | | 0.15 | | 0.10 | | | 0.01 | | 0.89 | | 0.26 | | <.01 | | | |  |  |
| 37. It bothered me that things did not go as expected | 0.31 | <.01 | | 0.25 | | 0.01 | | 0.17 | | 0.06 | | 0.28 | | <.01 | | 0.21 | | 0.02 | | 0.09 | | 0.30 | | 0.30 | | <.01 | | 0.09 | | 0.33 | | | <.01 | | 0.98 | | 0.34 | | <.01 | | | |  |  |
| 38. I was bored quickly | 0.05 | | 0.58 | | 0.08 | | 0.38 | | 0.06 | | 0.53 | | 0.14 | | 0.12 | | 0.10 | | 0.27 | | 0.10 | | 0.26 | | 0.01 | | 0.95 | | <.01 | | 0.98 | | | 0.10 | | 0.28 | | 0.12 | | 0.20 | | | | |
| 39. I felt impatient | 0.20 | | 0.03 | | 0.19 | | 0.04 | | 0.08 | | 0.35 | | 0.22 | | 0.02 | | 0.26 | | <.01 | | 0.09 | | 0.34 | | 0.20 | | 0.03 | | 0.18 | | 0.05 | | | 0.03 | | 0.78 | | 0.31 | | <.01 | |  |  |  |
| 40. I was unable to sit still | 0.14 | | 0.12 | | 0.05 | | 0.56 | | 0.04 | | 0.62 | | 0.14 | | 0.11 | | 0.25 | | <.01 | | 0.06 | | 0.51 | | 0.26 | | <.01 | | 0.30 | | <.01 | | | 0.03 | | 0.71 | | 0.22 | | 0.01 | |  |  |  |
| 41. I was impulsive | 0.03 | | 0.73 | | 0.04 | | 0.67 | | -0.01 | | 0.93 | | 0.08 | | 0.37 | | 0.18 | | 0.05 | | 0.09 | | 0.35 | | 0.20 | | 0.03 | | 0.30 | | <.01 | | | 0.06 | | 0.52 | | 0.20 | | 0.03 | |  |  |  |
| 42. I felt irritated | 0.26 | | <.01 | | 0.20 | | 0.03 | | 0.05 | | 0.59 | | 0.22 | | 0.01 | | 0.18 | | 0.05 | | 0.02 | | 0.82 | | 0.18 | | 0.05 | | 0.09 | | 0.34 | | | 0.02 | | 0.84 | | 0.34 | | <.01 | |  |  |  |
| 43. Others felt annoyed by me | 0.09 | | 0.34 | | 0.08 | | 0.41 | | 0.05 | | 0.57 | | 0.06 | | 0.53 | | 0.12 | | 0.20 | | -0.05 | | 0.60 | | 0.13 | | 0.15 | | 0.14 | | 0.12 | | | 0.04 | | 0.66 | | 0.19 | | 0.04 | |  |  |  |
| 44. I had a fight | -0.11 | | 0.22 | | 0.06 | | 0.52 | | -0.05 | | 0.61 | | -0.10 | | 0.27 | | -0.10 | | 0.26 | | -0.11 | | 0.24 | | -0.02 | | 0.83 | | 0.06 | | 0.48 | | | 0.07 | | 0.43 | | 0.06 | | 0.51 | |  |  |  |
| 45. I felt rebellious | 0.10 | | 0.25 | | 0.07 | | 0.47 | | 0.02 | | 0.84 | | 0.08 | | 0.37 | | 0.18 | | 0.05 | | -0.04 | | 0.64 | | 0.15 | | 0.10 | | 0.13 | | 0.14 | | | 0.02 | | 0.79 | | 0.20 | | 0.03 | |  |  |  |
| 46. Things did not bother me | 0.07 | | 0.48 | | 0.09 | | 0.35 | | 0.06 | | 0.51 | | 0.17 | | 0.05 | | 0.14 | | 0.13 | | 0.01 | | 0.87 | | 0.19 | | 0.03 | | 0.21 | | 0.02 | | | -0.02 | | 0.85 | | 0.18 | | 0.05 | |  |  |  |
| 47. I had moodswings | 0.23 | | 0.01 | | 0.20 | | 0.03 | | 0.17 | | 0.06 | | 0.25 | | 0.01 | | 0.25 | | 0.01 | | <.01 | | 0.99 | | 0.17 | | 0.07 | | 0.04 | | 0.69 | | | 0.05 | | 0.60 | | 0.29 | | <.01 | |  |  |  |
| 48. I felt strange | 0.20 | | 0.03 | | 0.12 | | 0.20 | | 0.14 | | 0.13 | | 0.22 | | 0.02 | | 0.21 | | 0.02 | | 0.06 | | 0.53 | | 0.11 | | 0.23 | | 0.08 | | 0.41 | | | 0.05 | | 0.55 | | 0.22 | | 0.02 | |  |  |  |
| 49. I felt suspicious | 0.18 | | 0.04 | | 0.15 | | 0.11 | | 0.11 | | 0.23 | | 0.21 | | 0.02 | | 0.14 | | 0.14 | | 0.01 | | 0.90 | | 0.05 | | 0.56 | | -0.04 | | 0.66 | | | 0.05 | | 0.62 | | 0.21 | | 0.02 | |  |  |  |
| 50. I experienced physical pain | 0.28 | | <.01 | | 0.29 | | <.01 | | 0.06 | | 0.55 | | 0.25 | | 0.01 | | 0.10 | | 0.27 | | 0.14 | | 0.13 | | 0.19 | | 0.03 | | 0.21 | | 0.02 | | | -0.01 | | 0.92 | | 0.22 | | 0.01 | |  |  |  |
| 51. I experienced physical discomfort | 0.22 | | 0.01 | | 0.32 | | <.01 | | 0.09 | | 0.30 | | 0.31 | | <.01 | | 0.12 | | 0.19 | | 0.15 | | 0.10 | | 0.08 | | 0.40 | | 0.09 | | 0.30 | | | 0.01 | | 0.90 | | 0.26 | | <.01 | |  |  |  |
| 52. I drank alcohol today | -0.13 | | 0.16 | | -0.01 | | 0.90 | | 0.03 | | 0.76 | | -0.20 | | 0.03 | | -0.02 | | 0.82 | | 0.01 | | 0.94 | | -0.12 | | 0.18 | | -0.01 | | 0.93 | | | 0.49 | | <.01 | | -0.10 | | 0.25 | |  |  |  |
| 53. I used (soft)drugs today | 0.05 | | 0.58 | | 0.15 | | 0.11 | | 0.24 | | 0.01 | | 0.15 | | 0.11 | | 0.11 | | 0.24 | | 0.25 | | 0.01 | | -0.04 | | 0.64 | | 0.03 | | 0.73 | | | 0.50 | | <.01 | | 0.10 | | 0.26 | |  |  |  |
| 54. Number of pleasant events | -0.21 | | 0.02 | | -0.23 | | 0.01 | | -0.20 | | 0.03 | | -0.23 | | 0.01 | | -0.05 | | 0.56 | | -0.11 | | 0.22 | | 0.05 | | 0.55 | | -0.10 | | 0.27 | | | -0.06 | | 0.52 | | -0.22 | | 0.02 | |  |  |  |
| 55. Intensity of pleasant events | 0.03 | | 0.73 | | <.01 | | 1.00 | | -0.09 | | 0.30 | | -0.10 | | 0.29 | | 0.10 | | 0.26 | | 0.02 | | 0.81 | | 0.13 | | 0.14 | | 0.06 | | 0.52 | | | -0.07 | | 0.45 | | <.01 | | 0.99 | |  |  |  |
| 56. Number of unpleasant events | 0.08 | 0.39 | | 0.08 | | 0.41 | | -0.02 | | 0.83 | | 0.17 | | 0.06 | | 0.05 | | 0.57 | | -0.05 | | 0.56 | | 0.18 | | 0.05 | | 0.07 | | 0.44 | | -0.06 | | | 0.52 | | 0.18 | | 0.04 | | | | |  |
| 57. Intensity of unpleasant events | 0.33 | <.01 | | 0.33 | | <.01 | | 0.12 | | 0.18 | | 0.35 | | <.01 | | 0.23 | | 0.01 | | 0.14 | | 0.11 | | 0.21 | | 0.02 | | 0.11 | | 0.21 | | -0.04 | | | 0.66 | | 0.40 | | <.01 | | | | |  |
| 58. I’m dreading something that will happen soon | 0.31 | <.01 | | 0.20 | | 0.03 | | 0.11 | | 0.23 | | 0.33 | | <.01 | | 0.21 | | 0.02 | | 0.08 | | 0.41 | | 0.19 | | 0.03 | | 0.01 | | 0.93 | | 0.06 | | | 0.48 | | 0.22 | | 0.01 | | | | |  |
